# Oridonin inhibits aberrant AKT activation in breast cancer

**DOI:** 10.18632/oncotarget.24378

**Published:** 2018-02-01

**Authors:** Bowen Sun, Geng Wang, Huidong Liu, Pian Liu, Waleed O. Twal, Hiuwing Cheung, Steven L. Carroll, Stephen P. Ethier, Emily E. Mevers, Jon Clardy, Thomas Roberts, Changbin Chen, Qian Li, Lanfeng Wang, Meixiang Yang, Jean J. Zhao, Qi Wang

**Affiliations:** ^1^ The first Affiliate Hospital, Biomedical Translational Research Institute, Guangdong Province Key Laboratory of Molecular Immunology and Antibody Engineering, Jinan University, Guangzhou 510632, China; ^2^ Department of Pathology and Laboratory Medicine, Medical University of South Carolina, Charleston, SC 29425, USA; ^3^ Department of Anatomy, Harbin Medical University, Harbin 150081, China; ^4^ Cancer Center, Union Hospital, Tongji Medical College, Huazhong University of Science and Technology, Wuhan 430000, China; ^5^ Department of Regenerative Medicine and Cell Biology, Medical University of South Carolina, Charleston, SC 29425, USA; ^6^ Department of Biological Chemistry and Molecular Pharmacology, Harvard Medical School, Boston, MA 02115, USA; ^7^ Department of Cancer Biology, Dana-Farber Cancer Institute, Boston, MA 02115, USA; ^8^ Institute Pasteur of Shanghai, Chinese Academy of Sciences, Shanghai 200031, China

**Keywords:** TCM plant extracts, oridonin, PI3K/AKT signaling, mammary tumor prevention, tumorigenesis

## Abstract

Aberrant activation of phosphatidylinosito-4,5-bisphosphate 3-kinase/protein kinase B (PI3K/AKT) signaling in cancer has led to pursuit of inhibitors for targeting this pathway. However, inhibitors of PI3K and AKT have failed to yield efficacious results without adverse effects. Here, we screened a library containing 441 authenticated traditional chinese medicine (TCM) plant extracts by examining their effect on cell viability of a human mammary epithelial cell line HMEC-PIK3CA^H1047R^, which expresses mutant PIK3CA^H1047R^ and has constitutively active AKT signaling. We found that Oridonin, an extract from *Rabdosia rubescens*, reduced cell viability to the greatest extent. Oridonin binds to AKT1 and potentially functions as an ATP-competitive AKT inhibitor. Importantly, Oridonin selectively impaired tumor growth of human breast cancer cells with hyperactivation of PI3K/AKT signaling. Moreover, Oridonin prevented the initiation of mouse mammary tumors driven by PIK3CA^H1047R^. Our results suggest that Oridonin may serve as a potent and durable therapeutic agent for the treatment of breast cancers with hyperactivation of PI3K/AKT signaling.

## INTRODUCTION

The lipid phosphatidylinositol 3,4,5-trisphosphate (PIP_3_) is a critical signaling molecule located at the cell membrane, the level of which is tightly regulated by the opposing activities of two enzymes: the phosphatidylinositol 3-kinase (PI3K; a lipid kinase) and phosphatase and tensin homologue (PTEN; a lipid phosphatase). Under normal physiological condition, growth factor stimulation of receptor tyrosine kinases (RTKs) activates PI3K that uses phosphatidylinositol 4,5-trisphosphate (PIP_2_) as a substrate to generate PIP_3_. Increased PIP_3_ levels result in the activation of the serine/threonine kinase AKT and downstream effector pathways that regulate many biological processes including cell proliferation and survival. The tumor suppressor PTEN functionally antagonizes PI3K activity via its intrinsic lipid phosphatase activity by converting PIP_3_ back to PIP_2_ [1, 2]. The PI3K/AKT signaling pathway is improperly activated in many types of human cancer by RTKs and somatic mutations in specific components of the signaling pathway [3]. In breast cancer, human epidermal growth factor 2 (HER2), a RTK, is constitutively activated by overexpression or gene amplification in 15–20% of breast tumors [4]. The *PIK3CA* gene, which encodes the p110α isoform of PI3K, is frequently mutated in more than 25% of breast cancer [5–8]. Loss of PTEN occurs in an additional of 25% of breast cancer [9, 10]. Genetic alterations of all three isoforms of AKT have also been observed in breast cancers [11, 12].

Many small molecule drugs targeting different isoforms or components of the PI3K/AKT signaling pathway are in clinical development. Some of these drugs inhibit all isoforms of the catalytic subunit of class IA PI3Ks (p110α, p110β, and p110δ) may cause undesired toxicity. Some inhibitors are designed to inhibit individual isoforms in cancers in which the activation of PI3K/AKT signaling relies on specific isoforms only. However, it remains unclear which type of inhibitor will be more effective clinically. Since feedback signaling has been shown to limit the efficacy of PI3K inhibitors [13], dual PI3K/mTOR inhibitors have also been developed. However, a critical issue that will influence the advantage of these inhibitors is whether complete inhibition of all isoforms of PI3K and mTORC1/2 will be tolerable in patients or whether the use of these inhibitors will necessitate sacrificing complete inhibition of one or more of the potential targets. There is still an urgent need for new inhibitors that effectively shut down PI3K/AKT signaling with minimum toxicity.

Medicinal plants, including those used in traditional chinese medicine (TCM), have historically proven their value as a source of molecules with therapeutic potential and represent an important pool for the identification of novel drugs. Here, we screened a library of authenticated TCM plants containing 441 plants extracts by the cell viability assay in a human mammary epithelial cell line HMEC-PIK3CA^H1047R^ with constitutively activated PI3K and AKT signaling. Of these, Oridonin, an extract from *Rabdosia rubescens*, reduced cell viability to the greatest extent. We demonstrated that Oridonin functioned as a potential ATP-competitive AKT inhibitor. Oridonin selectively impaired the growth of human breast cancer cells with hyperactivation of AKT signaling (p-AKT^High^) *in vitro* and *in vivo*. Our results suggest that Oridonin may be of substantial clinical utility in breast cancers with hyperactivation of PI3K/AKT signaling.

## RESULTS

### Screen of compounds in TCM plant extracts that inhibit the growth of HMEC- PIK3CA^H1047R^ cells

**Figure 1:**
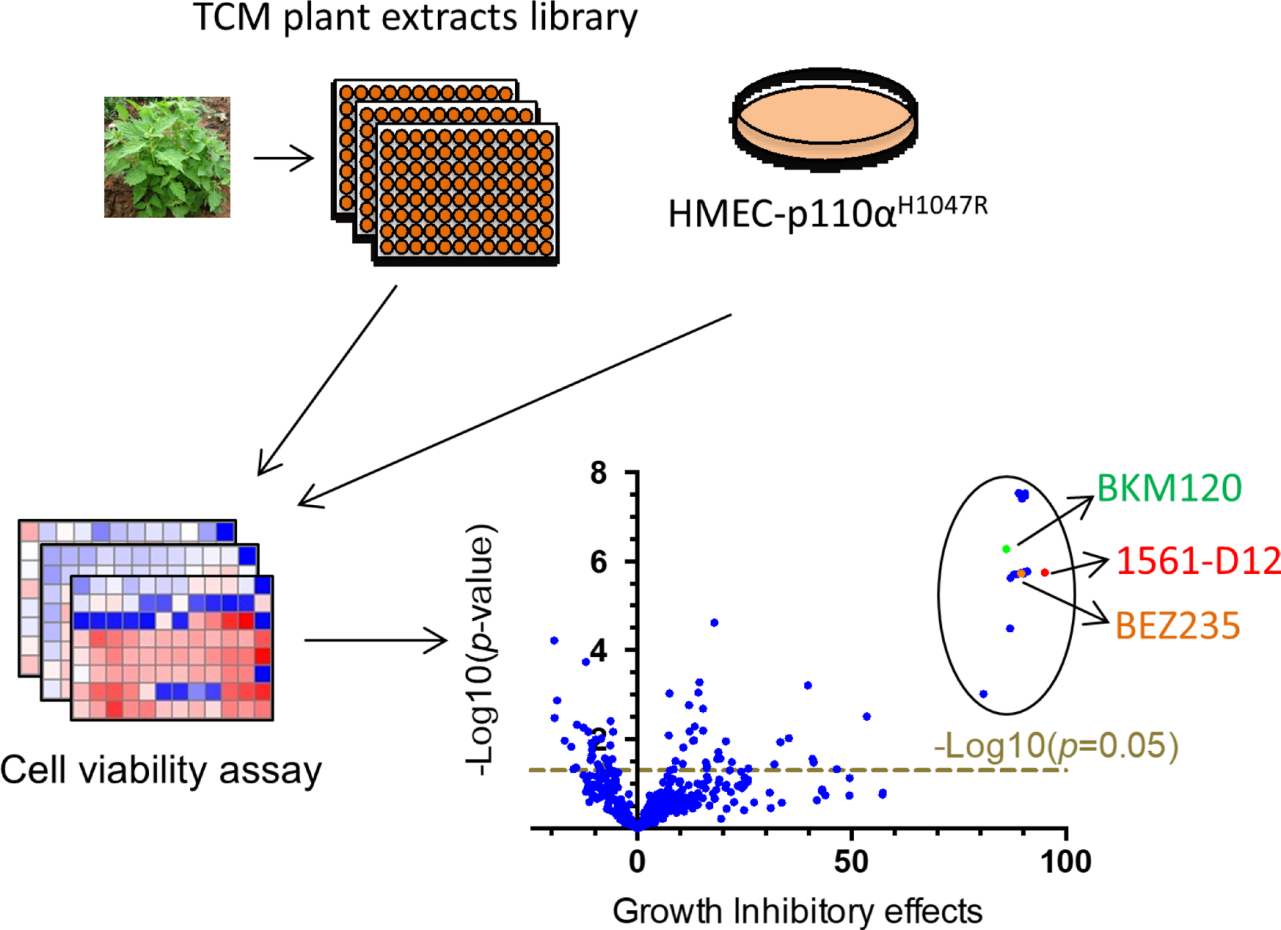
Schematic of the screen for TCM plant extracts HMEC-PIK3CA^H1047R^ cells with constitutively active ATK signaling were generated by introducing oncogenic mutant PIK3CA^H1047R^ into an immortalized human mammary epithelial cell (HMEC) cell line. The cells were seeded in 96-well plates and treated in triplicate with a TCM library containing 441 extracts from plants used in traditional Chinese medicine. After 3 days of exposure to 1.5 μg/mL of extracts, cell numbers were assessed using the CellTiter-Glo luminescent cell viability assay. Volcano plots were generated by plotting the growth inhibitory effects against probability. Candidate compounds are highlighted by the circle in the upper right corner.

To identify potential PI3K pathway inhibitors from TCM plant extracts we used a Celltiter-Glo cell viability assay to screen a library containing 441 partially purified plant extracts [14] in HMEC-PIK3CA^H1047R^ cells, which possess constitutively activated AKT signaling due to ectopic expression of mutant PIK3CA^H1047R^ in an immortalized human mammary epithelial cell (HMEC) line (Supplementary Figure 1). The results from the screen are represented in Figure 1 as volcano plots displaying the statistical significance against growth inhibition in the cells. We found that 54 out of 441 extracts significantly inhibited HMEC-PIK3CA^H1047R^ cell growth (*p <* 0.05) (Supplementary Table 1). Among them, 19 extracts, derived from *Rabdosia rubescens*, *Glycyrrhiza uralensis Fisch*, and *Sophora flavescens Ait*, showed more than 80% cell growth inhibition comparable to the effect of the pan PIK3 inhibitor BKM120 or the dual PI3K/mTOR inhibitor BEZ235 (Figure 1 and Supplementary Table 2). 1561-D12, an extract from *Rabdosia rubescens*, showed maximal growth inhibition (Figure 1 and Supplementary Table 2). Liquid chromatography-mass spectrometry (LC/MS) and nuclear magnetic resonance (NMR) analyses of 1561-D12 showed that the fraction contained a relatively pure major chemical entity, which was identified as Oridonin (Supplementary Table 3), a previously described compound with potential antitumor activity in a variety of cancer cells [15–19].

### Oridonin preferentially suppresses AKT/mTOR signaling in human mammary epithelial cells

**Figure 2:**
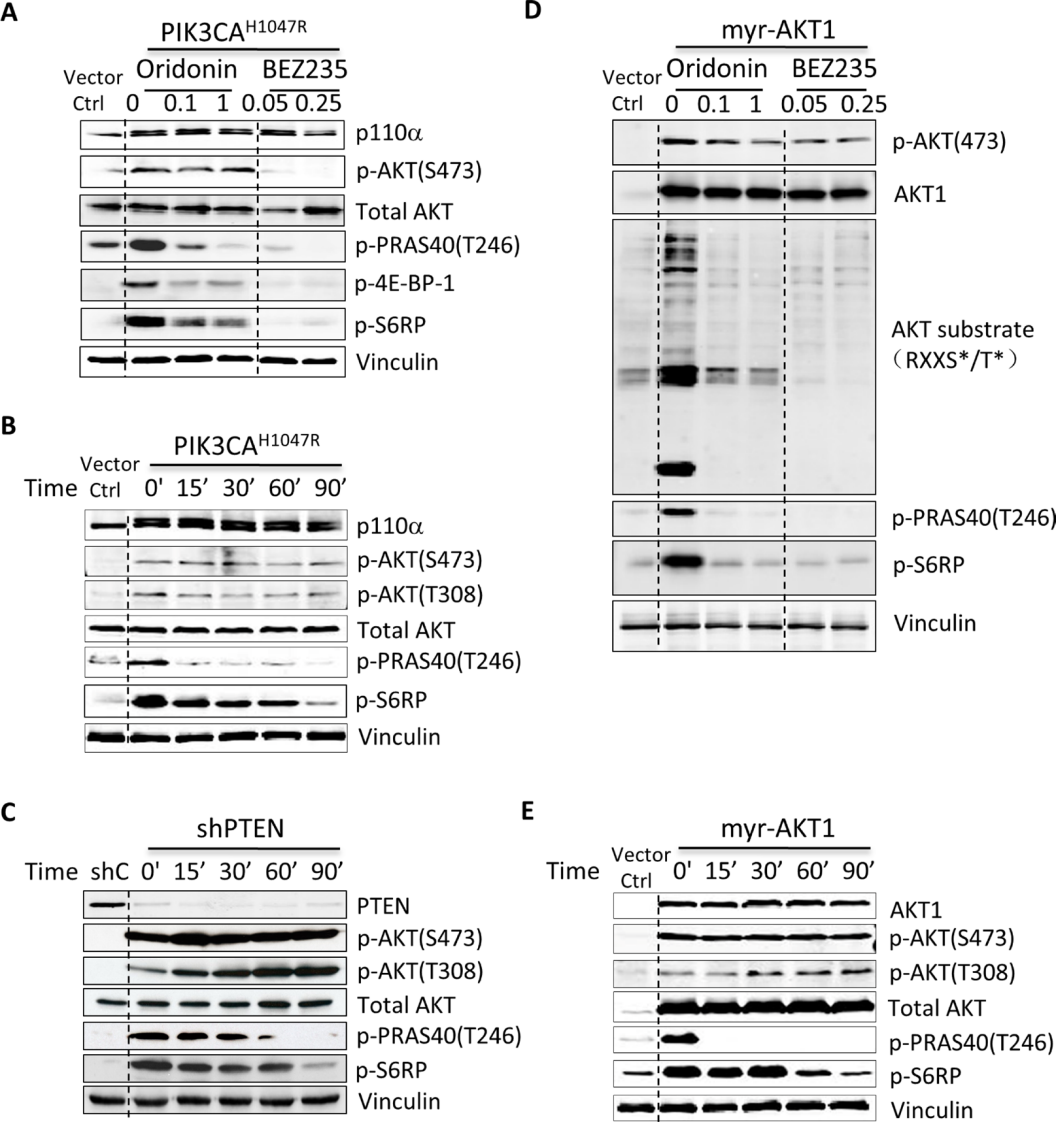
Oridonin preferentially suppresses AKT/mTOR signaling HMEC-PIK3CA^H1047R^ (**A**) or HMEC-myr-AKT1 (**D)** cells were starved for 2 hrs and then treated with vehicle control (DMSO) or increasing concentrations of Oridonin (0.1 and 1 μM) or BEZ235 (50 and 250 nM) for 1 hr before preparation of lysates for immunoblotting using the indicated antibodies. HMEC-PIK3CA^H1047R^ (**B)**, HMEC-shPTEN (**C**) and HMEC-Myr-AKT1 (**E)** cells were starved for 2 hrs and then treated with vehicle control (DMSO) or Oridonin (1 μM) for 0, 15, 30, 60, and 90 minutes before preparation of lysates for immunoblotting using the indicated antibodies.

We then investigated whether Oridonin suppressed PI3K signaling or activated AKT itself in HMEC-PIK3CA^H1047R^ cells. We performed immunoblotting to evaluate PI3K signaling components in HMEC-PIK3CA^H1047R^ cells after treatment with increasing concentrations of Oridonin or the dual PI3K/mTOR inhibitor BEZ235. Unexpectedly, Oridonin did not significantly suppress phosphorylation of AKT as BEZ235 did, but did efficiently inhibit the phosphorylation of AKT substrates, such as AKT substrate 1 (AKTS1; also known as PRAS40) and downstream mTOR signaling proteins (Figure 2A).

To further determine whether Oridonin suppresses PI3K signaling, we treated HMEC-PIK3CA^H1047R^ cells in a time-dependent manner. Consistent with the above data, Oridonin suppressed the phosphorylation of PRAS40 and S6RP at 15 minutes post treatment but did not inhibit the phosphorylation of AKT even after 90 minutes (Figure 2B). To determine whether Oridonin inhibited PI3K signaling in other cells with activation of AKT signaling, we stably depleted PTEN via shRNA in HMEC cells. PTEN protein expression was suppressed in stable pLKO-shPTEN [20] expressing HMEC cells, and phosphorylation of AKT and its effectors increased (Figure 2C). Consistent with the effect of Oridonin in HMEC-PIK3CA^H1047R^ cells, Oridonin suppressed the phosphorylation of PRAS40 and S6RP starting from 15 minutes post treatment, but had a minimal effect on phosphorylation of AKT (Figure 2C).

To investigate the broad effect of Oridonin in AKT specifically, we evaluated the spectrum of AKT phosphorylation substrates using phospho-AKT substrate-specific antibodies that recognize the phospho-(Ser/Thr) AKT substrate motif (RXXS*/T*) in an engineered cell line HMEC-myrAKT1 that ectopically expresses myristoylated AKT1. When directed to membranes by the addition of a src *myristoylation* sequence, AKT becomes constitutively active. Oridonin gradually suppressed pan-AKT phosphorylation substrates in HMEC-myrAKT1 cells comparable to the effects of the PI3K/mTOR dual inhibitor BEZ235 (Figure 2D), pan PI3K initiator BKM120, AKT inhibitor MK2206, and mTOR inhibitor RAD001 (Supplementary Figure 2). Moreover, as expected, Oridonin suppressed the phosphorylation of PRAS40 and S6RP starting from 15 minutes post treatment, but did not inhibit phosphorylation of AKT in these cells (Figure 2E). These results indicate that Oridonin preferentially blocks AKT kinase activity by inhibiting phosphorylation of AKT substrates and subsequently suppresses downstream mTOR signaling.

**Figure 3:**
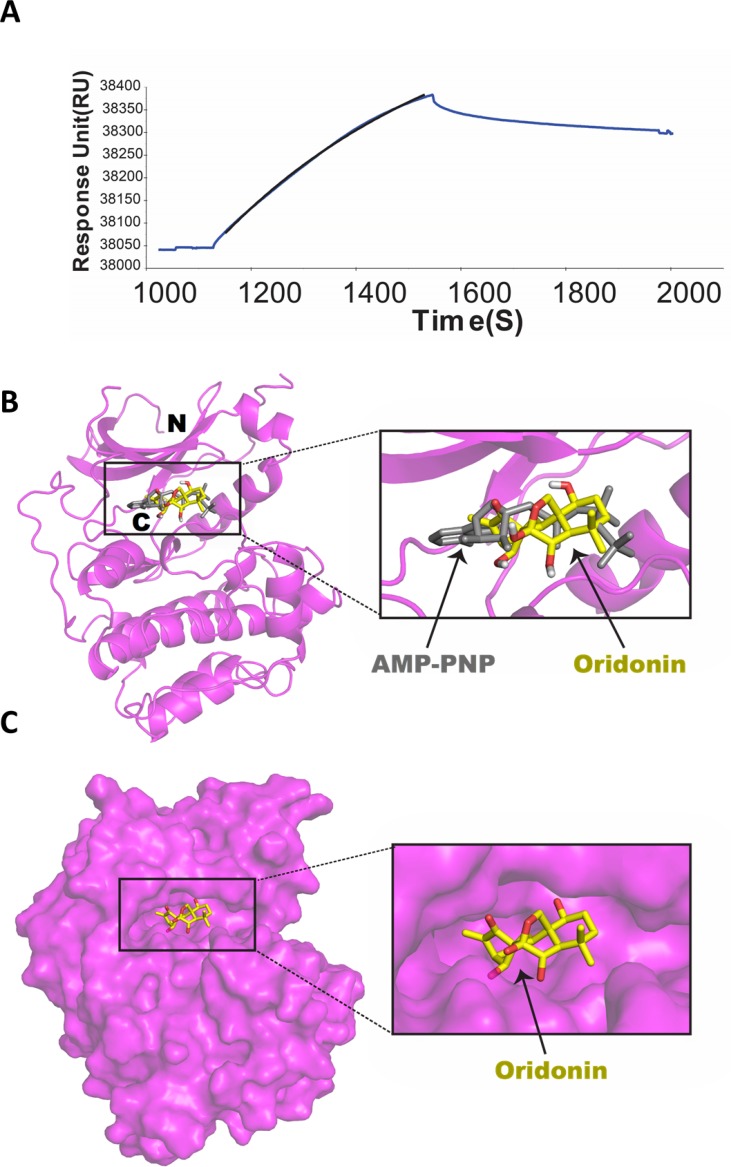
Surface plasmon resonance (SPR) analysis of Oridonin binding to AKT1 (**A**) SPR sensorgrams of Oridonin (2 μM) binding to purified human recombinant AKT1 on a sensor chip CM5 are shown. Data depicted were normalized to 100 response units (RU) and are representative of three independent experiments. To obtain affinity constants (KD), SPR profiles in a given series were simultaneously fit to a 1:1 binding site model using BIAevaluation Version 3.1 software. Original sensogram is shown in blue and best fit association line from BIA evaluation software is in black. (**B**) Docking model of Oridonin binding AKT1. Ribbon representation of overlaid Oridonin docking model with the complex structure of AKT1 with AMP-PNP (PDB ID: 4EKK; magenta, protein; gray, ligand). (**C**) Surface representation of Oridonin binding in the nucleotide-binding pocket. For oridonin, C_20_H_28_O_6_ (C, yellow; O, red) and hydrogens were hidden to simplify the 3D structure.

To investigate whether Oridonin directly binds to AKT, we performed surface plasmon resonance (SPR) analysis to evaluate the *in vitro* binding of Oridonin with purified human recombinant AKT1. As shown in Figure 3A, Oridonin bound to AKT1 immobilized on a sensor chip. Optimal fitting of SPR data obtained by measuring the binding of Oridonin at a concentration of 2 μM to immobilized AKT1 was best achieved using a single class binding site model. As a result, an affinity constant (KD) of 2.15 nM (*n* = 3; *χ*^2^ of fit < 10) was determined for Oridonin binding to AKT1. AKT protein isoforms (AKT1, 2 and 3) are structurally plastic enzymes, with large conformational differences observed between the inactive and the active kinase domain structures including order changes in the N-terminal pleckstrin homology (PH) domain, an interdomain linker, a kinase domain, and a C-terminal hydrophobic motif [21]. Using a well-documented docking software-AutoDock Vina due to its accuracy of the predictions of the experimental structure [22], we predict a docking model of Oridonin binding AKT1 (Figure 3B and 3C) based on the crystal structure of AKT1 complex with adenylyl-imidodiphosphate (AMP-PNP) [23], a non-hydrolysable analogue of ATP functions as an ATP-dependent competitive inhibitor. This model showed that Oridonin did bind AKT1 in the ATP binding pocket of the kinase domains, because it has been overlaid very well with AMP-PNP (Figure 3B). Additionally, Oridonin binds the entry site of the ATP binding pocket, suggesting it might prevent ATP access to the nucleotide-binding site (Figure 3C). The active structure of AKT1 in complex with AMP-PNP was reported to limit accessibility to the phosphorylation sites [23]. Taken together, these results suggest that Oridonin may function as a potential ATP-competitive AKT inhibitor.

### Oridonin selectively suppresses growth of breast cancer cells with hyperactivation of AKT signaling

**Figure 4:**
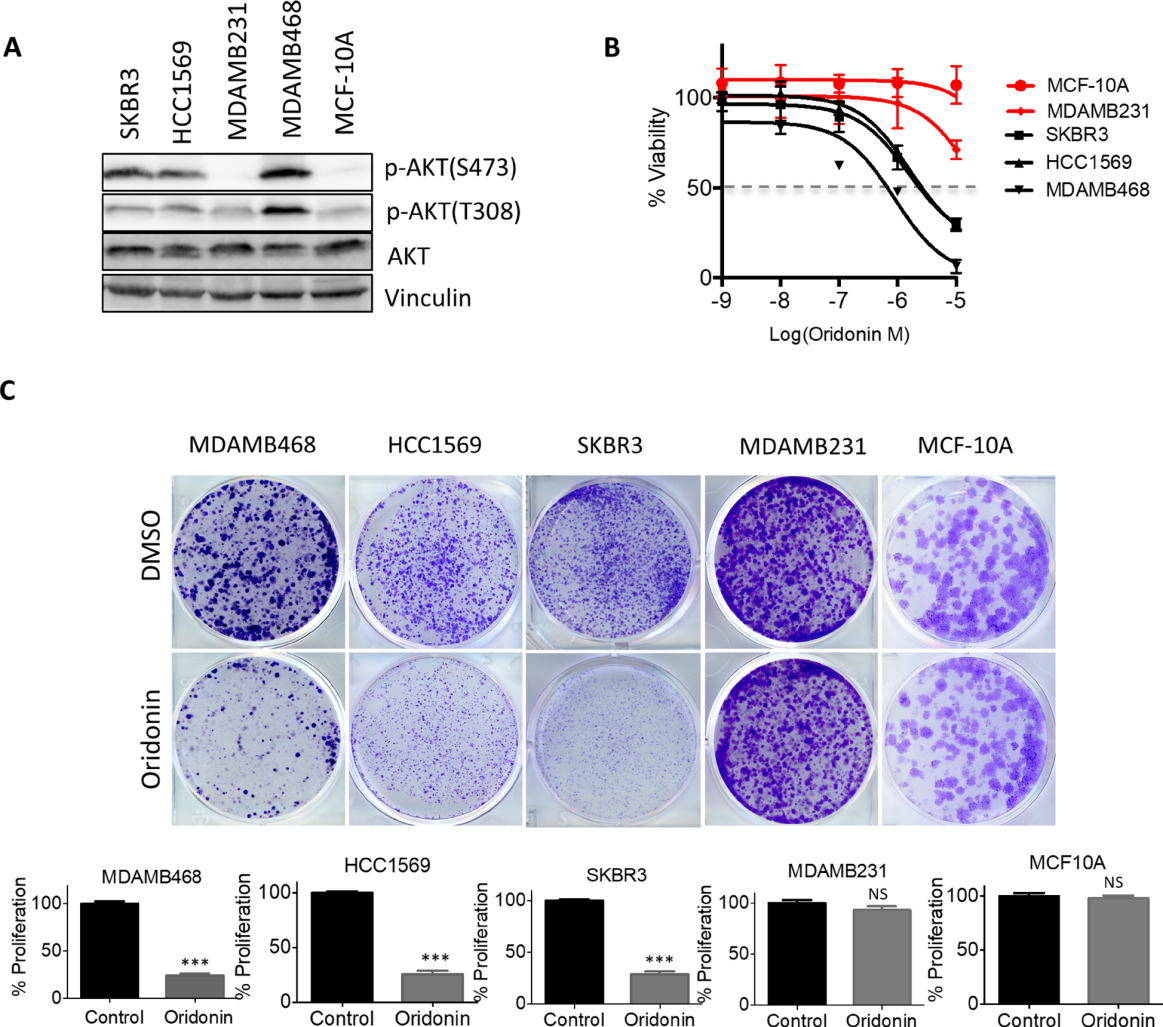
Oridonin selectively impairs cell growth and tumorigenesis in breast cancer with hyperactivation of AKT signaling (**A**) Immunoblot analysis of AKT phosphorylation levels in breast cancer cell lines. (**B**) Dose-response curve of breast cancer cells after treatment with increasing concentrations of Oridonin for 72 hrs. Percent viability relative to that of DMSO-treated cells is shown. Data represent mean ± SD of three replicates. (**C**) Crystal violet staining of cells and quantification of cell growth. Data are presented as mean ± SD of three replicates, ^*^p < 0.01, ^***^p < 0.001 (Student’s *t* test).

To investigate whether Oridonin inhibits the cell viability of other breast cancer cells we determined dose-response curves in a panel of breast cancer cell lines with different levels of phosphorylated AKT (Figure 4A). Three breast cancer cells had high levels of AKT phosphorylation (p-AKT^High^) due to activation by HER2 amplification (SKBR3 and HCC1569) or PTEN loss (MDAMB468), whereas MDAMB231 and the immortalized mammary epithelial cell MCF-10A expressed low levels of p-AKT. p-AKT^High^ cells were more sensitive to Oridonin treatment, with cell viability effectively suppressed by 10-to 100-fold lower concentrations of Oridonin compared with MDAMB231 and MCF-10A cells (Figure 4B and Supplementary Figure 3). To investigate whether the proliferation of these cells is inhibited by Oridonin treatment, we performed clonogenic growth assays using the above cell lines. Cells were treated with 1 μM Oridonin for three weeks and stained with crystal violet. Consistent with the above findings, Oridonin selectively inhibited the clonogenic growth of p-AKT^High^ cells (MDAMB468, SKBR3, and HCC1569), but did not suppress proliferation of p-AKT^low^ MDAMB231 and MCF-10A cells (Figure 4C). These findings suggest that Oridonin selectively inhibited clonogenic growth of breast cancer cells with hyperactivation of AKT.

### Oridonin impairs growth of breast tumor with hyperactivation of AKT *in vivo*

**Figure 5:**
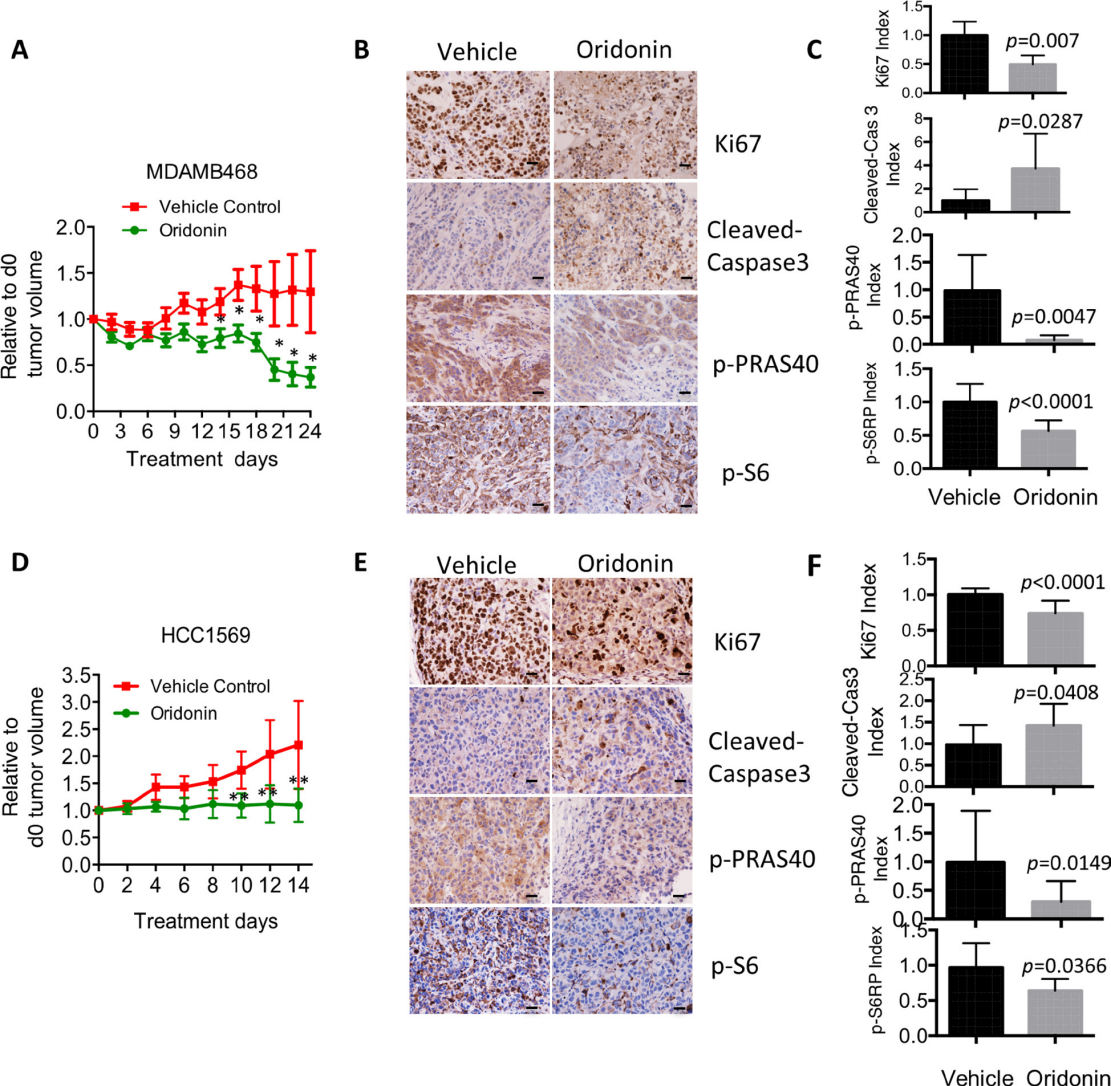
Oridonin impairs *in vivo* cell growth in breast cancer with hyperactivation of AKT signaling **(A**) Growth of triple negative breast cancer cells (MDAMB468) in nude mice treated with vehicle (Tn = 7) or Oridonin (Tn = 8, 15 mg/kg) by intraperitoneal injection. Mean ± SD values are presented. ^*^p < 0.05 (Student’s *t* test). (**B**) Growth of HER2-positive breast cancer cells (HCC1569) in nude mice treated with vehicle (Tn = 6) or Oridonin (Tn = 10, 15 mg/kg) by intraperitoneal injection. Mean ± SD values are presented. ^**^p < 0.01 (Student’s *t* test). (**C**, **D)** Immunohistochemical (IHC) analysis of AKT pathway, proliferation (Ki67), and apoptosis (cleaved caspase 3) in tumors harvested from animals treated with vehicle or Oridonin for 3 days. Scale bar represents 20 um. (**E**, **F**) Quantitative analyses of 6 IHC images randomly obtained from three mice. *p* values were assessed by Student’s *t* test.

To test whether Oridonin can impair the *in vivo* growth of p-AKT^High^ breast cancer cells, NCr nude mice bearing palpable MDAMB468 or HCC1569 xenografts were treated with Oridonin or vehicle control. Durable tumor regression was achieved in both MDAMB468 and HCC1569 xenograft tumor models following Oridonin treatment (Figure 5A and 5D). To evaluate signaling and pharmacodynamic responses of MDAMB468 and HCC1569 xenografts during Oridonin treatment, tumors were isolated 72 hrs after drug administration and molecular markers were analyzed by immunohistochemical staining. Oridonin decreased phosphorylation of the AKT substrate PRAS40 and AKT downstream mTOR target (S6), blocked proliferation (as assessed by Ki67 index), and induced apoptosis (as assessed by cleaved caspase 3) (Figure 5B, 5C, 5E, and 5F). These results indicate that Oridonin effectively impairs tumor growth in p-AKT^High^ breast cancers by inhibiting proliferation and inducing apoptosis via suppressing AKT-mTOR signaling pathway.

### Oridonin prevents the initiation of mammary tumors carrying PIK3CA^H1047R^ by blocking AKT-mTOR signaling

**Figure 6:**
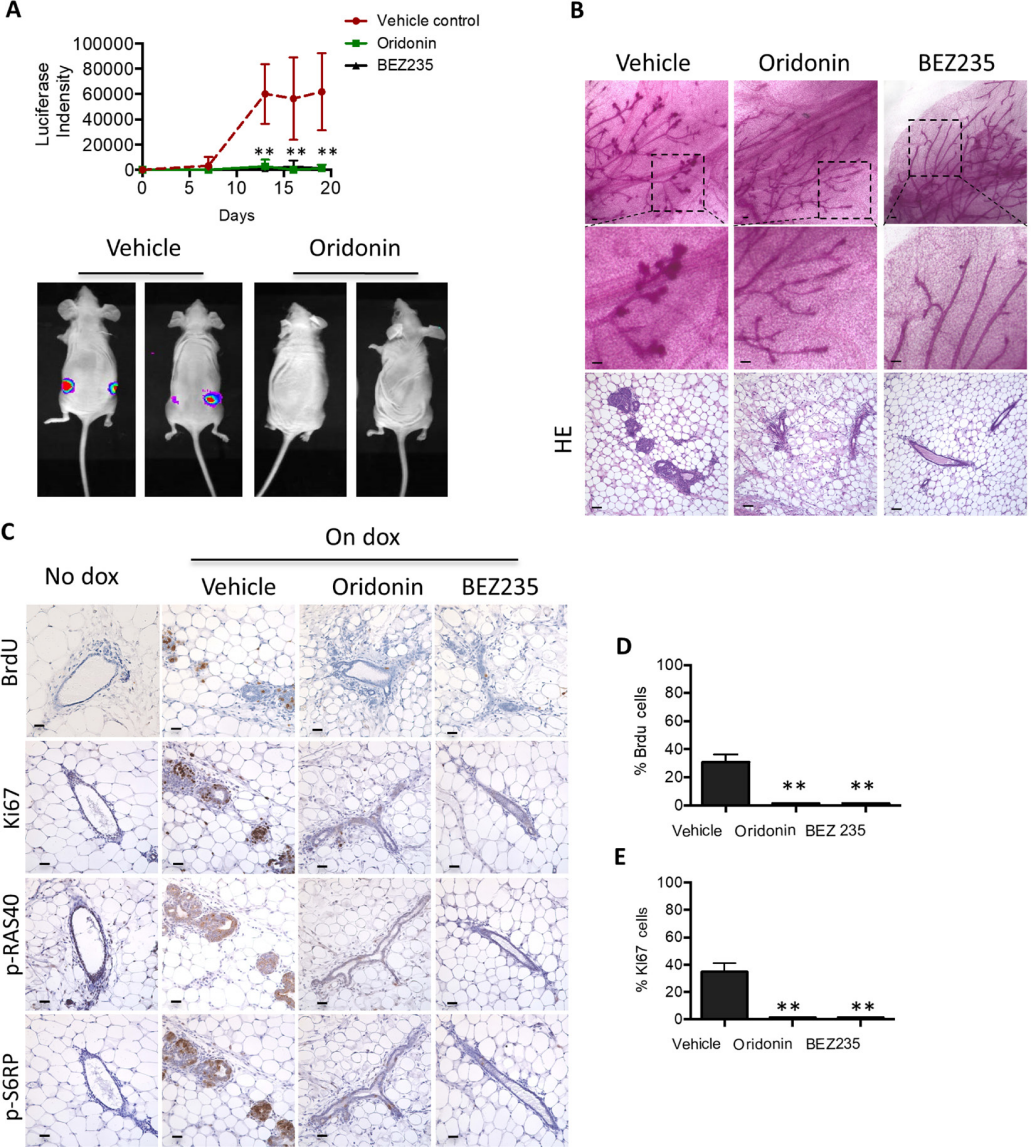
Oridonin prevents initiation of mouse mammary tumors carrying PIK3CA_H1047R_ (**A**) Mammary glands from bitransgenic mice MMTV-rtTA-tetO-PIK3CAH1047R were transplanted into NCr nude mice. Doxycycline was introduced into the diet 8 weeks after transplantation. Intensities of luciferase were analyzed in mice bearing doxycycline-inducible PIK3CAH1047R mammary glands after treatment with vehicle (*n* = 9), Oridonin (*n* = 9) or BEZ235 (*n* = 4) (top panel). ^*^p < 0.01, ^**^p < 0.001 (Student’s *t* test). Representative images at the time of the last treatment are shown (bottom panel). (**B**) Mammary gland whole mounts prepared from mice at the end of treatment with vehicle, Oridonin, or BEZ325, Scale bars represent 20 um. **(C–E)** Immunohistochemical analysis of proliferation, assessed by Brdu and Ki67 staining, and AKT signaling in tumors harvested from animals treated with vehicle, Oridonin, or BEZ325 at 18 days. Scale bars represent 20 um. Quantitative analyses of 6 images randomly obtained from three mice. ^*^p < 0.01, ^**^p < 0.001 (Student’s *t* test).

We previously reported that expression of PIK3CA^H1047R^ could initiate transformation of mammary epithelium in inducible MMTV-rtTA-tetO-PIK3CA^H1047R^ (iPIK3CA^H1047R^) female mice [24]. To examine whether Oridonin can prevent PIK3CA^H1047R^-induced mammary epithelial cell transformation, we transplanted PIK3CA^H1047R^ mammary tissue fragments into cleared fat pads of 3-week-old NCr nude female mice. PIK3CA^H1047R^ expression in iPIK3CA^H1047R^ mammary epithelial cells is coupled to a luciferase reporter, allowing transgene expression to be followed *in vivo* [24]. Expression of PIK3CA^H1047R^ in transplanted mammary epithelial cells was induced by treating the mice with doxycycline at 8 weeks post transplantation. Mice were concurrently treated with Oridonin, BEZ235 or vehicle control. The increased luciferase reporter activity in transplanted iPIK3CA^H1047R^ epithelium induced by doxycycline treatment was blocked by treatment with Oridonin and BEZ235 (Figure 6A). Histological examination showed increased mammary ductal side-branching and enlarged focal nodular structures filled with hyperproliferative cells characteristic of early neoplastic lesions in the vehicle control group whereas normal mammary epithelium structures were observed in mice treated with Oridonin or BEZ235 (Figure 6B). Immunohistochemical analyses showed that Oridonin significantly eliminated AKT effector phosphorylation and blocked cell proliferation in the iPIK3CA^H1047R^ mammary outgrowths (Figure 6C–6E). These results establish that Oridonin prevents cell transformation by blocking AKT and downstream mTOR signaling in response to the induction of PIK3CA^H1047R^.

## DISCUSSION

In this study, we screened a library of 441 TCM plant extracts by examining their effect on cell viability in a HMEC-PIK3CA^H1047R^ cell model with constitutively active AKT signaling. Nineteen of the extracts impaired cell growth as efficiently as the pan PI3K inhibitor BKM120 or dual PI3K/mTOR inhibitor BEZ235. We showed that Oridonin, an extract from *Rabdosia rubescens*, reduced cell viability most efficiently. Rather than inhibit PI3K activity directly, Oridonin bound to AKT1 and may function as a potential ATP-competitive AKT inhibitor. Oridonin selectively impaired the cell growth of p-AKT^High^ human breast cancers *in vitro* and *in vivo* by preferentially blocking AKT-mTOR signaling. Oridonin also efficiently prevented the initiation of mouse mammary tumors driven by PIK3CA^H1047R^ *in vivo*. Our results suggest that Oridonin may serve as a potent and selective therapeutic agent in patients bearing breast cancers with hyperactivation of AKT signaling.

Although the popularity of herbal therapies has increased globally in recent years, their efficacy, safety, mechanisms of action, and potential as novel therapeutic agents remain poorly defined. *Rabdosia rubescens* and its extracts were shown to suppress disease progression, reduce tumor burden, and prolong survival in patients with esophageal cancer, gastric carcinoma, or liver cancer [15–18]. The antitumor activity of Oridonin, a bitter tetracycline diterpenoid compound isolated from *Rabdosia rubescens*, was demonstrated in many types of cancers including prostate, breast, non-small cell lung cancers, acute leukemia, glioblastoma, and human melanoma [15–19]. However, the mechanisms underlying the antitumor activity of Oridonin remain largely unknown. Here, we showed for the first time that Oridonin directly bound to AKT1 and may function as a potential ATP-competitive AKT inhibitor. Oridonin impaired cell proliferation and induced apoptosis in a p-AKT dependent manner. Oridonin effectively inhibited the phosphorylation of pan-AKT substrates and subsequently blocked downstream effector mTOR signaling in breast tumor cells with HER2 amplification/overexpression (SKBR3 and HCC1569), PTEN loss (MDAMB468, HMEC-shPTEN), mutant PI3K (HMEC-PIK3CA^H1047R^, mouse mammary epithelium with PIK3CA^H1047R^), and AKT1 overexpression (HMEC-myr-ATK1) (Figures 2 and 4), suggesting broad therapeutic benefits of Oridonin in breast cancer cells with hyperactivation of AKT signaling. Importantly, breast cancer cells without AKT activation (MDAMB231) were less sensitive to Oridonin, with IC50 values 10 times higher than those in cells with AKT activation. The non-transformed cell line MCF10A was far less sensitive to Oridonin, with IC50 value 100 times higher than that in breast cancer cells with AKT activation (Supplementary Figure 3), leading to potent and selective cytotoxicity while sparing normal cells. The selective cytotoxicity of Oridonin in p-AKT^High^ breast cancer cells provides a therapeutic advantage that cannot be achieved by the pan PI3K inhibitor BKM120, dual PI3K/mTOR inhibitor BEZ235, AKT inhibitor MK2206 (Supplementary Figure 4). In view of potential AKT-independent signaling, which exhibits only minimal AKT activation, downstream of oncogenic PI3KCA mutation in human cancers [25], the phosphorylation level of AKT should be considered as a therapeutic marker of Oridonin treatment. Notably, Oridonin efficiently prevented the initiation of mouse mammary tumors with PI3KCA mutant cells (Figure 6) suggesting the potential application and advantages of *Rabdosia rubescens* or its extracts for carriers of breast cancer susceptibility genes who are at high risk of developing breast cancer; for example, carriers of germline PTEN mutation or BRAC1 mutation show increased AKT signaling [26–28]. In addition to Oridonin, another 18 extracts from *Glycyrrhiza uralensis Fisch* or *Sophora flavescens Ait* inhibited the growth of HMEC-PIK3CA^H1047R^ cells by more than 80% compared to cells treated with DMSO (Supplementary Table 1), suggesting the potential role of other TCM plant extracts in inhibition of PI3K signaling and cancer therapy.

Our results provide concrete evidence that *Rabdosia rubescens* extract Oridonin decreases AKT signaling and selectively inhibits tumor growth in p-AKT^High^ breast tumors cells. Of note, to date pan-or isoform-selective inhibitor of PI3K inhibitors, AKT inhibitors, mTOR inhibitors, and dual PI3K/mTOR inhibitors have not yielded durable or efficacious clinical results, Oridonin offers promise of both increased efficacy and reduced toxicity compared with single PI3K, AKT, or even dual PI3K/mTOR inhibitors. Together, our data suggest that in the context of p-AKT^High^ breast cancers, Oridonin may serve as a potent and durable therapeutic agent and should be considered for clinical application.

## MATERIALS AND METHODS

### TCM plant extract library

The production of the plant extract library was obtained by the ICCB-Longwood Screening Facility at Harvard Medical School (https://iccb.med.harvard.edu). The library contains 441 extracts from plants used in traditional Chinese medicine. Crude extracts from the plants were obtained and further fractionated to eliminate nuisance compounds, especially those of high molecular weight or high polarity [14].

### LC/MS and NMR techniques for compound characterization

HPLC purifications were carried out using an Agilent 1200 series HPLC system equipped with a photo-diode array detector and a Phenomenex 4 µm Hydro semi-preparative column. Compounds eluted at minute 25 when run using the following gradient: holding 20% ACN + 0.1% formic acid (FA)/H_2_O + 0.1% FA for 5 min, followed by a shallow gradient to 40% ACN + 0.1% FA/H_2_O + 0.1% FA over 30 min and then fast gradient to 100% ACN + 0.1% FA over 5 min. LR-LCMS data was obtained using an Agilent 1200 series HPLC system equipped with a photo-diode array detector and a 6130 quadrupole mass spectrometer. NMR spectra were recorded with pyridine as an internal standard (δ_H_ 8.71, 7.58, and 7.22; δ_C_ 150.4, 135.9, and 123.9) on a Varian Oxford 600 MHz spectrometer equipped with a 5 mm AutoX HCN triple res inverse probe. Compounds were identified either using authentic samples or comparing their chemical shifts with those reported in the literature [14].

### Cell culture

MDAMB468 and MDAMB231 cells were cultured in 10% fetal bovine serum (FBS) DMEM with penicillin/streptomycin. SKBR3 and HCC1569 cells were cultured in 10% FBS RPMI1640 with penicillin/streptomycin. HMEC-PIK3CA^H1047R^, HMEC-shPTEN, and HMEC-myr-AKT1 cells were cultured in 2.5% FBS DMEM/F12 supplemented with insulin, epidermal growth factor, and hydrocortisone, as described previously [29].

### Cell viability assay

Cell viability was assessed using a CellTiter-Glo luminescent cell viability assay (Promega). Equal numbers of cells were plated in 96-well plates and treated with serial dilutions of inhibitors for 72 hrs. Two independent experiments were performed per cell line, each with triplicate measurements.

### Western blotting

Cells were lysed in 1% NP-40 buffer supplemented with protease and phosphatase inhibitors (Roche). Equal amounts of proteins were resolved by SDS-PAGE and transferred to nitrocellulose membranes for western blotting. The primary antibodies used were specific for the following proteins: p-PRAS40-T246 (Invitrogen), p-AKT S473, p-AKT T308, p-S6 S240/244, p-4EBP1, p110a, PTEN, AKT1, p-AKT substrate (RXXS*/T*), and AKT (all from Cell Signaling Technology). Secondary anti-mouse and anti-rabbit IgG fluorophore conjugated antibodies were used to visualize the indicated proteins on an Odyssey scanner (Li-Cor).

### Kinetic analysis of oridonin-AKT1 binding

Surface plasmon resonance (SPR) measurements of the interaction of Oridonin with purified human recombinant AKT1 protein (Abcam) were performed on a BIAcore 3000 instrument. AKT1 was immobilized on BIAcore sensor chips (type CM5, GE Healthcare Life Sciences) at 14450 RU using an amine coupling kit. Oridonin was dissolved in 1% Pluronic F68 (Sigma) detergent by sonication at 1 mg/mL. Running buffer used in the experiment was HEPES/NaCl buffer containing 1% Pluronic F68 detergent at a flow of 20 μl/minute Oridonin (diluted to 2 μM) was injected for 7 minutes over the chip surface in a Biacore 3000 machine and then allowed to dissociate for a further 7 minutes. The binding analysis was performed using BIA evaluation software.

### Histology and immunohistochemistry (IHC)

Formalin-fixed paraffin-embedded tissue sections were prepared by the Biorepository & Tissue Analysis Shared Resource, Hollings Cancer Center. BrdU labeling and staining were done according to the manufacturer’s instructions (BD Biosciences). IHC was performed with antibodies against Ki67 (Vector labs), p-PRAS40-T246 (Invitrogen), p-S6 (Cell Signaling Technology), and cleaved-caspase 3 (Cell Signaling Technology). For quantification of IHC, 6 images were taken per tumor section and analyzed using the Visiopharm Integrator System (Visiopharm).

### Xenograft studies

All animal experiments were conducted in accordance with animal use guidelines of the National Institutes of Health using protocols approved by the Medical University of South Carolina and the Dana-Farber Cancer Institute Animal Care and Use Committee. Breast cancer cells were harvested and resuspended in 40% Matrigel-Basement Membrane Matrix, LDEV-free (BD Biosciences), and then injected (100 μl per site) into the fourth pair of mammary fat pads of nude mice (CrTac: NCr-Foxn1nu). Tumors were measured in two dimensions using manual calipers. Tumor volume was calculated using the formula: Volume = 0.5 × length × width × width. Tumor volume was measured every 2–3 days. Upon harvesting, tumors were fixed in formalin overnight and then in 70% ethanol for histopathology analysis.

### Inhibitor administration

Animals were treated with Oridonin (15 mg/kg; Chengdu Purification Technology Development Co., Ltd, Chuengdu, China) in 1% Pluronic F68 (Sigma) or vehicle (1% Pluronic 68) daily by intraperitoneal (IP) injection as described previously [19]. BEZ235 (Haoyuan Chemo express Co. Ltd, Shanghai, China) was reconstituted 1:9 in 1-methyl-2 pyrolidone (NMP; Sigma) and polyethylene glycol 300 (PEG300; Fluka Analytical). Mice were treated with this compound formulation at 45 mg/kg daily (QD) by oral gavage.

### Mammary fragment transplantation

Mammary glands were isolated from 8-week-old female MMTV-rtTA-tetO-PIK3CA^H1047R^ mice [24]. Mammary gland fragments were transplanted into cleared fat pads of 3-week-old NCr nude female mice as described previously [30]. Doxycycline induction was performed 8 weeks post-transplantation by introduction of a 2500 ppm doxycycline containing diet [31].

### Bioluminescence imaging

Mice were anesthetized with ketamine and xylazine and administered D-luciferin (Promega) IP to monitor luciferase gene expression *in vivo*. Images were analyzed using KODAK Molecular Imaging Software (version 4.5.0b6 SE).

### Mammary gland whole mounts

Transplanted mammary glands were harvested and mounted on glass slides. Slides were fixed in an ethanol-glacial acid mixture and stained with carmine red, followed by dehydration and a final clearing in toluene as described previously [30].

### Statistical analyses

Statistical analysis was performed as described for each experiment. All data are presented as mean ± s.d. Student's *t*-test (two-tailed) was used to compare two groups (*p* < 0.05 was considered significant) for independent samples.

## SUPPLEMENTARY MATERIALS FIGURES AND TABLES


