# Supplementary material for: Oridonin inhibits aberrant AKT activation in breast cancer
Source: Oncotarget. 2018 Feb 1;9(35):23878–89. doi: 10.18632/oncotarget.24378 (PMC5963618; doi:10.18632/oncotarget.24378)
Supplement: Supplementary file 1 [file oncotarget-09-23878-s001.pdf]

Oridonin inhibits aberrant AKT activation in breast cancer

SUPPLEMENTARY MATERIALS

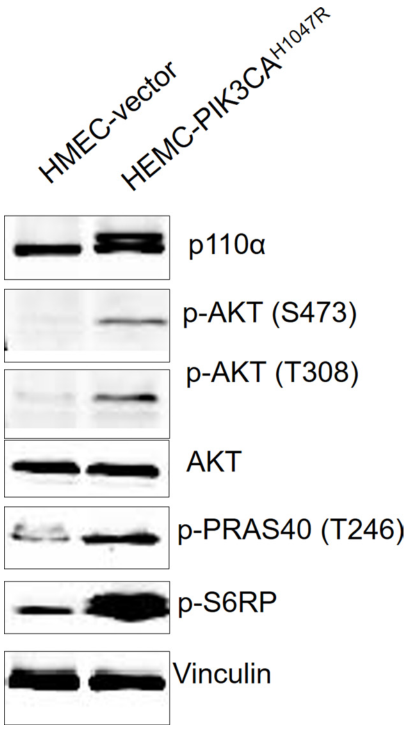

Supplementary Figure 1: Constitutively active ATK signaling in HMEC-PIK3CA^{H1047R} cells. Immunoblot analysis of AKT signaling in PIK3CA^{H1047R} and vector control HMEC cells.

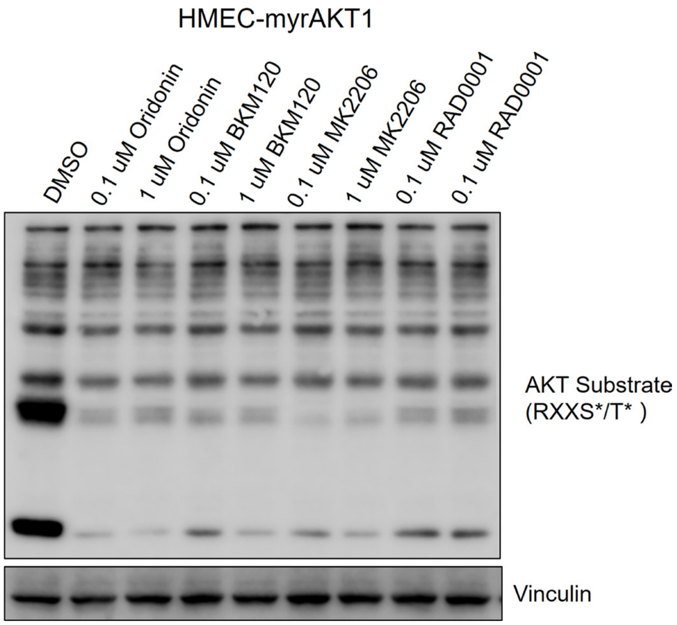

Supplementary Figure 2: Oridonin and inhibitors of PI3K signaling inhibited AKT phosphorylation of substrates in HMEC-myr-AKT1 cells. HMEC-myr-AKT1 cells were starved for 2 hrs and then treated with vehicle control (DMSO) or increasing concentrations of Oridonin (0.1 and 1 μM), pan PI3K inhibitor BKM120 (0.1, 1 μM), AKT inhibitor MK2206 (0.1 and 1 μM), and mTOR inhibitor RAD 001 (0.1 and 1 μM) for 1 hr before preparation of lysates for immunoblotting.

Cell line	IC50 (M)
MDAMB468	1.76+0.58e-006
SKBR3	1.24±0.29e-006
HCC1569	2.08±0.36e-006
MDAMB231	2.29±0.17e-005
MCF-10A	1.13±0.63e-004

Supplementary Figure 3: IC50 of Oridonin in different breast cancer cells. Dose-response curve of breast cancer cells after treatment with increasing concentration of Oridonin for 72 hrs. IC50 was calculated by Prism software.

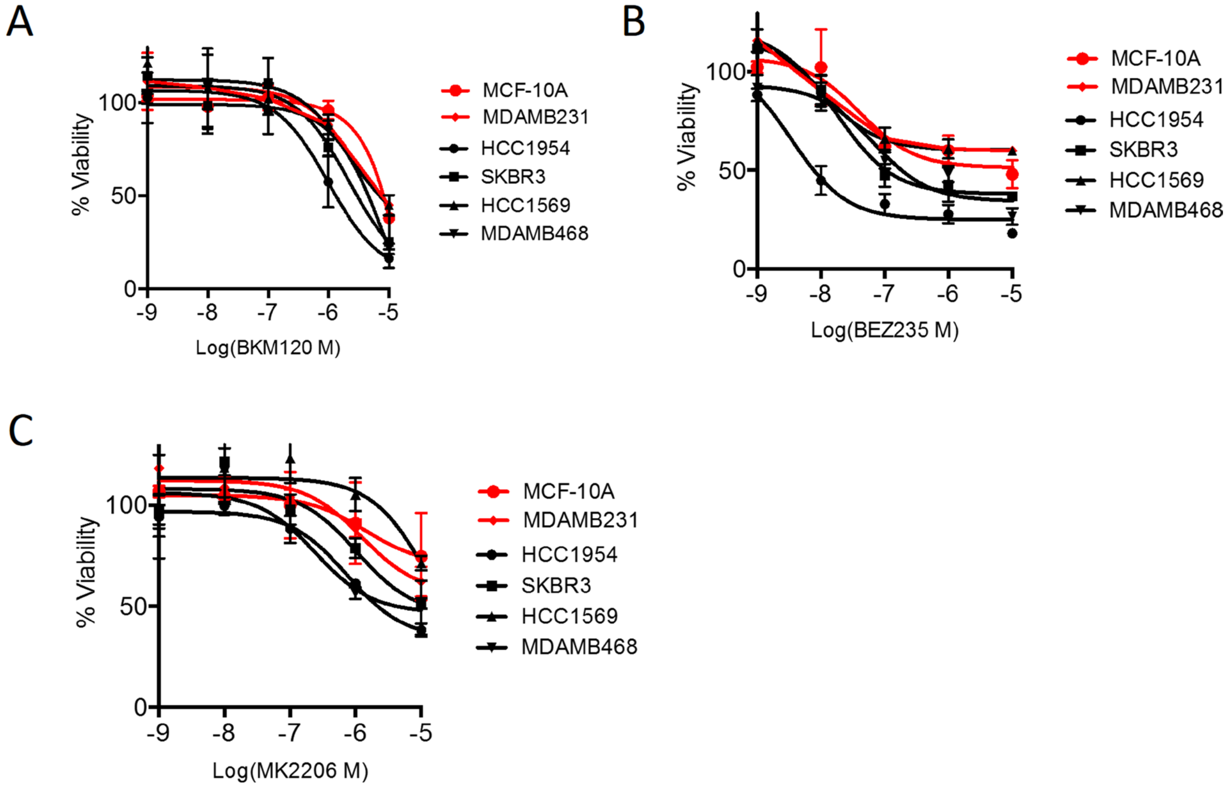

Supplementary Figure 4: Dose-response curve of breast cancer cells treated with inhibitors of PI3K signaling pathway. Dose-response curve of breast cancer cells after treatment with increasing concentration of BEZ235, BKM120, MK2206 for 72 hrs. Percent viability relative to that of DMSO-treated cells is shown. Data represent mean ± SD of three replicates.

Supplementary Table 1: Growth inhibition of TCM plant extracts, $p < 0.05$

Well name	Ave. Growth inhibition effects %	*p*-value
1561-D12	95.08589912	1.79413E-06
1952QA1-C5	91.01267711	1.70143E-06
1952QA1-C4	90.60014323	1.76695E-06
1952QA2-C11	90.55097559	3.01828E-08
1952QA2-C10	90.51335649	3.40147E-08
1952QA1-B9	90.48668451	1.75592E-06
1952QA1-C2	90.40793358	1.77451E-06
1952QA1-C3	90.39113811	1.7833E-06
1952QA1-B10	90.18520836	1.76862E-06
1952QA1-B11	90.07701442	1.78475E-06
1952QA1-C1	89.8135505	1.79995E-06
1952QA2-C9	89.81223803	3.94008E-08
1952QA2-C5	89.10055785	3.02546E-08
1952QA2-C4	89.05105663	2.95752E-08
1952QA1-B6	88.62545714	1.94859E-06
1952QA1-B5	87.88642538	1.99981E-06
1952QA1-B8	87.12119139	2.34782E-06
1952QA1-C7	87.00316798	3.2068E-05
1952QA2-C8	80.81947631	0.000950925
1952QA1-G7	53.56946931	0.003049659
1952QB1-D7	46.5824382	0.046398135
1952QA1-B4	41.16254863	0.032755581
1952QA2-H7	40.87872925	0.027526832
1952QA2-E8	39.78806026	0.000610194
1952QB2-D5	35.40712829	0.009397239
1952QB2-D6	33.36971559	0.011586976
1952QB2-D7	31.96186936	0.036177004
1952QB2-D4	25.85103834	0.044770003
1952QB2-F7	22.00749453	0.032436459
1952QB2-F6	21.52653877	0.049469625
1952QB1-C5	20.6406278	0.011078691
1952QB2-F5	19.40992698	0.02684136
1952QB1-C6	18.86936131	0.019059941
1952QB2-F8	18.59166878	0.027424264
1952QA2-D5	17.94423825	2.38933E-05
1952QB1-C8	16.24417855	0.039984347
1952QA1-F7	16.11402044	0.044107842
1952QA2-F6	15.92491909	0.032720637
1952QA2-E6	15.28644959	0.002039101
1952QB1-E7	15.26239909	0.006374243
1952QA2-E7	14.43025329	0.000516542
1952QA2-D6	14.24878059	0.000890718
1952QA2-F7	13.3844247	0.005121803
1952QA2-F5	13.23065747	0.010498083
1952QB1-E6	12.93503112	0.010616094
1953QA1-A8	12.13841748	0.006557752
1952QA2-D4	12.0711112	0.001685382
1952QA1-H6	10.71282187	0.015046681
1952QB1-E5	10.56078454	0.035254585
1952QA1-H7	8.868612171	0.030648154
1952QA1-H8	8.430134137	0.046721
1952QA1-H9	7.565747551	0.049992217
1952QA2-H5	7.443453287	0.000923024
1953QA1-A7	7.258163315	0.008135298

Supplementary Table 2: TCM Candidate extracts list

No.	Well name	Growth inhibition %	*p* value	Chinese name	Botanical name
1	1561-D12	95.08589912	1.79413E-06	Donglingcao	*Rabdosia rubescens*
2	1952QA1-C5	91.01267711	1.70143E-06	Gancao	*Glycyrrhiza uralensis Fisch*
3	1952QA1-C4	90.60014323	1.76695E-06	Gancao	*Glycyrrhiza uralensis Fisch*
4	1952QA2-C11	90.55097559	3.01828E-08	Kushen	*Sophora flavescens Ait.*
5	1952QA2-C10	90.51335649	3.40147E-08	Kushen	*Sophora flavescens Ait.*
6	1952QA1-B9	90.48668451	1.75592E-06	Gancao	*Glycyrrhiza uralensis Fisch*
7	1952QA1-C2	90.40793358	1.77451E-06	Gancao	*Glycyrrhiza uralensis Fisch*
8	1952QA1-C3	90.39113811	1.7833E-06	Gancao	*Glycyrrhiza uralensis Fisch*
9	1952QA1-B10	90.18520836	1.76862E-06	Gancao	*Glycyrrhiza uralensis Fisch*
10	1952QA1-B11	90.07701442	1.78475E-06	Gancao	*Glycyrrhiza uralensis Fisch*
11	1952QA1-C1	89.8135505	1.79995E-06	Gancao	*Glycyrrhiza uralensis Fisch*
12	1952QA2-C9	89.81223803	3.94008E-08	Kushen	*Sophora flavescens Ait.*
13	1952QA2-C5	89.10055785	3.02546E-08	Kushen	*Sophora flavescens Ait.*
14	1952QA2-C4	89.05105663	2.95752E-08	Kushen	*Sophora flavescens Ait.*
15	1952QA1-B6	88.62545714	1.94859E-06	Gancao	*Glycyrrhiza uralensis Fisch*
16	1952QA1-B5	87.88642538	1.99981E-06	Gancao	*Glycyrrhiza uralensis Fisch*
17	1952QA1-B8	87.12119139	2.34782E-06	Gancao	*Glycyrrhiza uralensis Fisch*
18	1952QA1-C7	87.00316798	3.2068E-05	Gancao	*Glycyrrhiza uralensis Fisch*
19	1952QA2-C8	80.81947631	0.000950925	Kushen	*Sophora flavescens Ait.*
PI3k/mTOR inhibitor	BEZ235	90.12970829	1.89829E-06	N/A	N/A
Pan PI3K inhibitor	BKM120	86.12965	5.30024E-07	N/A	N/A

Supplementary Table 3: 1561-D12 identification

Substance/Hit	Retention time	Mass (LC/MS)
Rabdosia (1561-D12)	12.27 min	257, 279
Oridonin	12.24 min	257, 279
